# ﻿Parazoanthidae (Cnidaria, Zoantharia) associated with glass sponges on the Nishi-Shichito Ridge, northwestern Pacific Ocean, with the description of a new species

**DOI:** 10.3897/zookeys.1221.131258

**Published:** 2024-12-27

**Authors:** Hiroki Kise, James Davis Reimer, Akira Iguchi, Yuji Ise, Shinji Tsuchida, Yoshihiro Fujiwara

**Affiliations:** 1 Geological Survey of Japan, National Institute of Advanced Industrial Science and Technology, AIST Tsukuba Central 7, 1-1-1 Higashi, Tsukuba, Ibaraki 305-8567, Japan; 2 Molecular Invertebrate Systematics and Ecology Laboratory, Graduate School of Engineering and Science, University of the Ryukyus, 1 Senbaru, Nishihara, Okinawa 903-0213, Japan; 3 Tropical Biosphere Research Center, University of the Ryukyus, 1 Senbaru, Nishihara, Okinawa 903-0213, Japan; 4 Research Laboratory on Environmentally-conscious Developments and Technologies (E-code), National Institute of Advanced Industrial Science and Technology (AIST), Tsukuba 305-8567, Japan; 5 Kuroshio Biological Research Foundation, 560 Nishidomari, Otsuki, Hata, Kochi 788-0333, Japan; 6 Research Institute for Global Change (RIGC), Japan Agency for Marine-Earth Science and Technology (JAMSTEC), 2-15 Natsushima-cho, Yokosuka, Kanagawa 237-0061, Japan

**Keywords:** Baseline data, Hexasterophora, mitochondrial genome, mitogenome, MPA, phylogeny, seamount, taxonomy, zoantharian

## Abstract

Seamounts are biodiversity hotspots that face increasing threats from anthropogenic activities. Seamounts host diverse sessile suspension-feeding organisms such as sponges and anthozoans, which are crucial for seamount ecosystems as they construct three-dimensional habitats utilized by numerous other animals. Therefore, accurate identification of seamount fauna, in particular of sessile suspension-feeding organisms, is of paramount importance for robust conservation efforts. This study focused on Zoantharia, a sessile anthozoan group, and specifically the family Parazoanthidae, known for associations with many different host taxa, prominently including octocorals and sponges. We collected Parazoanthidae specimens from northwestern Pacific seamounts and formally describe a new species, *Vitrumanthusflosculus* Kise & Reimer, **sp. nov.**, based on morphological and molecular analyses. We also report the complete mitochondrial genomes of this new species and the related species *Churabanakuroshioae.* Our results reconfirm the phylogenetic positions of these two species within Parazoanthidae, while demonstrating much remains to be learned about the benthic diversity of northwestern Pacific seamounts.

## ﻿Introduction

Seamounts are diversity hotspots for deep-sea organisms (Worm et al. 2003; Samadi et al. 2006; Clark et al. 2010; Morato et al. 2010; Rowden et al. 2010), harboring diverse assemblages of sessile suspension-feeding organisms due to turbulent and hydrodynamic water flowing around their peaks, which delivers planktonic food and nutrients to the benthos in the immediate area (Clark et al. 2010; Watling and Auster 2017). Seamount habitats and their fauna face threats from anthropogenic activities, such as bottom trawling that may damage or destroy these diverse marine animal forests (Worm et al. 2006; Clark et al. 2007; Althaus et al. 2009; Rossi et al. 2022). Sessile suspension-feeding organisms, especially sponges and anthozoans, play important roles in seamount communities, as they construct three-dimensional habitats utilized by numerous other animals such as crustaceans, ophiuroids, and polychaetes (Glasby and Watson 2001; Buhl-Mortensen and Mortensen 2004; Mosher and Watling 2009; Watling et al. 2011; Bracken-Grissom et al. 2018; Okanishi and Mah 2020; Komai et al. 2022). Sponges and anthozoans are vulnerable to damage from anthropogenic activities, as they are often large, fragile, long-lived, and extremely slow-growing (Probert et al. 1997; Clark et al. 2016; Molodtsova and Opresko 2017). It has been estimated that the recovery of these organisms from such anthropogenic damage will take decades to centuries (Clark et al. 2016). Therefore, accurate identification and documentation of seamount fauna, in particular of sessile suspension-feeding organisms, is important to generate robust baseline datasets that can be utilized to better protect the biological communities of seamounts.

The order Zoantharia is a group of sessile cnidarians consisting of > 300 species (Reimer and Sinniger 2024). In the deep sea, species of zoantharians within the family Parazoanthidae are known to associate with many different host taxa, prominently including octocorals and sponges (e.g., Carlgren 1923; Sinniger et al. 2005, 2013; Reimer et al. 2008, 2019; Carreiro-Silva et al. 2017; Kise et al. 2022; Montenegro et al. 2024). Four zoantharian genera have been reported to be associated with Hexasterophora sponges; *Churabana* Kise, Montenegro & Reimer, 2021, *Parachurabana* Kise, 2023, *Thoracactis* Gravier, 1918, and *Vitrumanthus* Kise, Montenegro & Reimer, 2022. *Churabana*, *Parachurabana*, and *Thoracactis* are monotypic genera while *Vitrumanthus* includes three species from the Pacific and Atlantic oceans. Although the Hexasterophora-zoantharian association thus has a wide distribution across the global oceans (Reiswig and Wheeler 2002; Dohrmann et al. 2011; Reiswig and Dohrmann 2014; Van Soest et al. 2014; Montenegro et al. 2020; Kise et al. 2022, 2023), the diversity of these associations is still poorly known. In this study, we collected specimens of *Churabana* and *Vitrumanthus* from the Shoho and An’ei seamounts along the Nishi-Shichito Ridge in the northwestern Pacific Ocean, and formally describe one species, *Vitrumanthusflosculus* sp. nov., utilizing a combination of morphological observations and molecular phylogenetic analyses. In addition, we report the complete mitochondrial genomes of two Hexasterophora-associated species, *Churabanakuroshioae* and *Vitrumanthusflosculus* sp. nov., which further reinforce the phylogenetic position of these species within Parazoanthidae.

## ﻿Materials and methods

### ﻿Specimen collection

**Figure 1.**
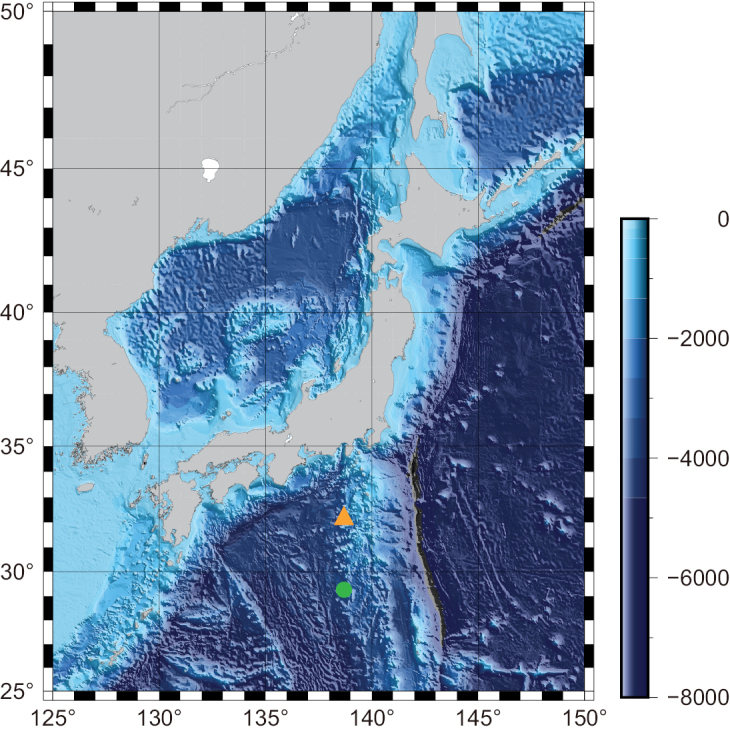
Research area and location of sampling sites. Enclosed symbols indicate sampling sites of two species examined in this study. *Vitrumanthusflosculus* sp. nov. (orange triangle) and *Churabanakuroshioae* (green circle).

Hexasterophora-associated zoantharians were collected from Shoho and An’ei seamounts on 29 November 2020 and 17 October 2021 by the remotely operated vehicle (*KM-ROV*) aboard the R/V Kaimei at depths of 400 and 770 m, respectively (Fig. 1). Photographs of the specimens were taken in situ for gross external morphological observation before collection using a camera mounted on the *KM-ROV*. Upon specimen retrieval, each specimen was anesthetized with magnesium chloride and subsequently fixed in 10% seawater formalin with subsamples preserved in 99.5% ethanol. The specimens examined in this study have been deposited in the National Museum of Nature and Science, Tsukuba, Japan (**NSMT**).

### ﻿DNA extraction, sequencing, and assembly

Tissues preserved in 99.5% ethanol were used for DNA extraction with a spin-column DNeasy Blood and Tissue Extraction kit following the manufacturer’s instructions (Qiagen, Hilden, Germany). Extracted DNA was quantified using a Qubit dsDNA BR assay kit (ThermoFisher Scientific, Waltham, USA). Whole-genome shotgun sequencing was performed by Bioengineering Lab. Co., Ltd. (Sagamihara, Japan) with DNBSEQ-G400 platforms (MGI Tech, Shenzhen, China) to produce pair-end 200 bp reads. The raw reads were filtered using Trimmomatic v. 0.39 (Bolger et al. 2014) with default parameters. Filtered reads were de novo assembled with GetOrganelle v.1.7.5 (Jin et al. 2020), which used implemented SPAdes v.3.6.2 genome assembler (Bankevich et al. 2012) with K-mer = 115. The mitochondrial genome annotation was performed with MITOS webserver (Bernt et al. 2013), and manually inspected and adjusted using Geneious Prime 2022.1.1 (https://www.geneious.com). Transfer RNA genes were identified using the tRNAscan-SE v2.0 (Chan et al. 2021). The annotated mitochondrial genomes were deposited in GenBank with the accession numbers PQ554681 and PQ554682. Sequences of Cox1 (mitochondrial cytochrome *c* oxidase subunit I), 12S rDNA (mitochondrial 12S ribosomal DNA), and 16S rDNA (mitochondrial 16S ribosomal DNA) were extracted from newly obtained mitochondria genomes. Three nuclear sequences, 18S rDNA (nuclear 18S ribosomal DNA), ITS rDNA (nuclear internal transcribed spacer region of ribosomal DNA), and 28S rDNA (nuclear 28S ribosomal DNA) were recovered from filtered and trimmed reads according to reference fragment sequences of *Churabanakuroshioae* (Accession numbers: MK377416, MZ329753, and MZ329743) and *Vitrumanthusschrieri* (Accession numbers: MZ329701, MZ329735, and MZ329712) using the Geneious Read Mapper (https://www.geneious.com).

### ﻿Molecular phylogenetic analyses

Partial fragments of mitochondrial genes (Cox1, 12S rDNA, and 16S rDNA) and of the nuclear genes (18S rDNA, ITSrDNA, and 28S rDNA) were added to the alignment dataset used in Kise et al. (2023). In addition, previously reported sequences of *Thoracactistopsenti* (Kise et al. 2024) were also added to the alignment dataset. GenBank accession numbers used for phylogenetic analyses in this study are listed in Suppl. material 1. Subsequently, these sequences were manually trimmed and realigned using MAFFT (Katoh and Standley 2013) with the auto algorithm under default parameters for all genetic markers, and finally these alignments for each genetic marker were concatenated (hereafter six-gene dataset). Phylogenetic analyses were performed on the concatenated dataset using maximum likelihood (ML) and Bayesian inference (BI). ModelTest-NG v.0.1.6 (Darriba et al. 2020) under the Akaike information criterion was used to select the best-fitting model for each molecular marker independently for both ML and BI analyses. The best-selected models for ML and BI analyses are listed in Suppl. material 2. The final dataset consisted of 5148 bp and was used for ML and BI analyses. ML analyses were performed by RAxML-NG (Kozlov et al. 2019) with 1000 bootstrap replicates. BI analyses were performed with MrBayes; four Markov chain Monte Carlo (MCMC) heated chains were run for 5,000,000 generations with the temperature of the heated chain set to 0.2. Chains were sampled every 200 generations. Burn-in was set to 1,250,000 generations, at which point the average standard deviation of split frequency was consistently below 0.01. Tracer v.1.7.1 (Rambaut et al. 2018) was used to inspect the convergence of MCMC.

In addition, 13 protein-coding genes were extracted from newly sequenced mitochondrial genomes and other zoantharian mitochondrial genomes listed in Poliseno et al. (2020) and Fourreau et al. (2023) (Suppl. material 3). These protein-coding genes were individually aligned using MAFFT with the auto algorithm under default parameters. The concatenated dataset consisted of 35 zoantharian species and 13025 sites. For this mitochondrial genome dataset, ML reconstruction was performed using IQ-TREE2 (Minh et al. 2020) with best-fitting models for each protein-coding gene selected using ModelFinder (Kalyaanamoorthy et al. 2017) implemented in the IQ-TREE2 under Bayesian information criterion (Suppl. material 4). Support for each node was evaluated using 10,000 ultrafast bootstrap (UFBoot2) approximations (Hoang et al. 2018). BI was performed with MrBayes v.3.2.7 (Ronquist et al. 2012); four Markov chain Monte Carlo (MCMC) heated chains were run for 5,000,000 generations with the temperature of the heated chain set to 0.2. Chains were sampled every 200 generations. Best-fitting models for BI analyses were selected from models available in MrBayes using IQ-TREE2 (-mset mrbayes) (Suppl. material 4). Burn-in was set to 1,250,000 generations, at which point the average standard deviation of split frequency was consistently below 0.01. Tracer v.1.7.1 (Rambaut et al. 2018) was used to inspect the convergence of MCMC. The mitochondrial genomes of two antipatharian species, *Stichopathesluetkeni* Brook, 1889 and *Myriopathesjaponica* (Brook, 1889), were used as outgroups according to Poliseno et al. (2020).

### ﻿Morphological observations

External morphological characteristics were observed and dissected under a Stemi 305 microscope (Carl Zeiss, Oberkochen, Germany), and photographs were taken using a Zeiss Axiocam 208 color camera (Carl Zeiss, Oberkochen, Germany). In addition, in-situ photographs were used for morphological observations. Internal morphological characters were examined by histological sections; 10–15-mm thickness serial sections were made with a microtome (Leica RM2145, Leica Biosystems, Wetzlar, Germany) and stained with haematoxylin and eosin after desilication with 20% hydrofluoric acid for 18–24 h. Classification of marginal muscle shapes followed Swain et al. (2015). Cnidae analyses were conducted using undischarged nematocysts and spirocysts from tentacles, column, actinopharynx, and mesenterial filaments using a Nikon Eclipse80i stereomicroscope (Nikon, Tokyo, Japan), and photographs were taken by a Nikon DS-Qi2 (Nikon, Tokyo, Japan). Cnidae sizes were measured using ImageJ v.1.45s (Rasband 2012). The reported frequencies were the relative amounts based on numbers from all slides in the cnidae analyses. Cnidae classification generally followed England (1991) and Ryland and Lancaster (2004) except for the treatment of basitrichs and microbasic b-mastigophores as in Kise et al. (2019).

## ﻿Results

### ﻿Taxonomic account

**Order Zoantharia Rafinesque, 1815**

**Suborder Macrocnemina Haddon & Shackleton, 1891**

**Family Parazoanthidae Delage & Hérouard, 1901**

#### ﻿Genus Vitrumanthus

Taxon classificationAnimalia

Kise, Montenegro & Reimer, 2022

06E67587-080B-56D6-8189-5AEABE104B5B

##### Type species.

*Vitrumanthusschrieri* Kise, Montenegro & Reimer, 2022.

##### Diagnosis.

Parazoanthidae characterized by obligate symbiotic relationship with massive hexasterophoran and Demospongiae sponges. Preserved polyps 0.3–3.1 mm in length, 0.8–3.4 mm in diameter. Azooxanthellate. Cyclically transitional marginal musculature.

#### ﻿ Vitrumanthus flosculus

Taxon classificationAnimalia

Kise & Reimer sp. nov.

79CE76E9-E591-5693-91B4-28BAD1978DAE

https://zoobank.org/BD579CA0-C245-4CBD-85DA-389A18CBAD7E

Figs 2 , 3 , 4 , 5

##### Material examined.

***Holotype*** • NSMT-Co 1898, Shoho Seamount, Nishi-Shichito Ridge, Japan (32°19.73'N, 138°44.28'E), 400 m depth, November 29, 2020.

##### Type locality.

Shoho Seamount, Nishi-Shichito Ridge, Japan.

##### Etymology.

“*flosculus*” meaning “small flower” or “floweret” in Latin.

##### Description.

**Figure 2.**
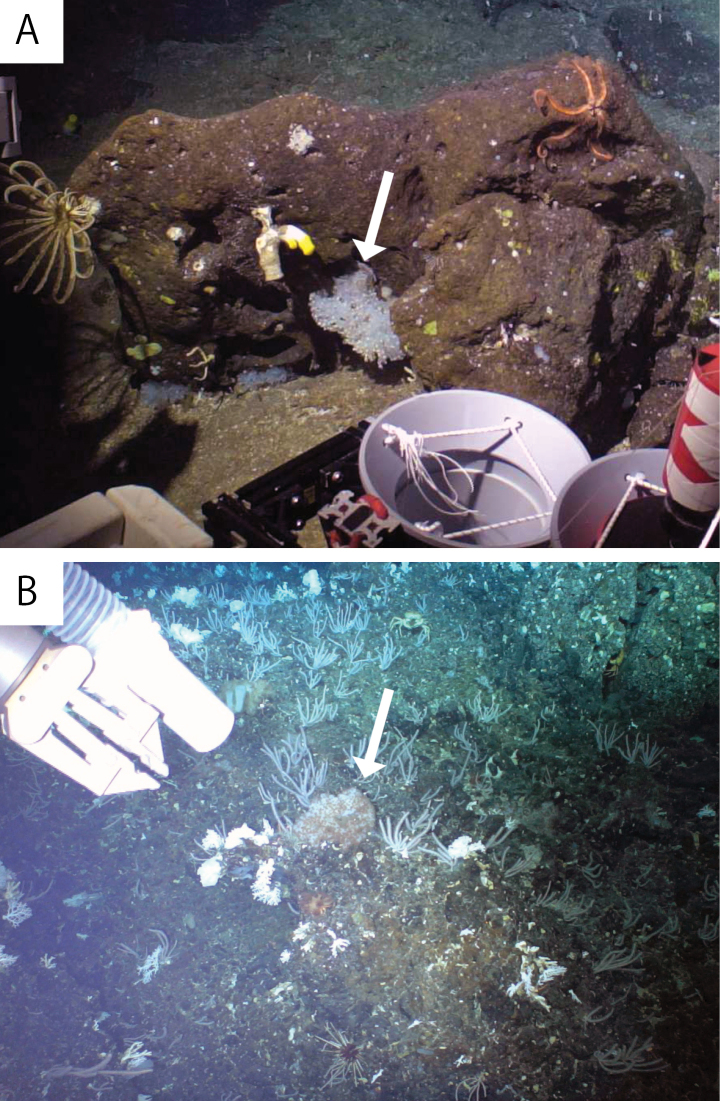
In-situ image of **A***Vitrumanthusflosculus* sp. nov. and **B***Churabanakuroshioae*. White arrows indicate each species associated with Hexasterophora sponges.

**Figure 3.**
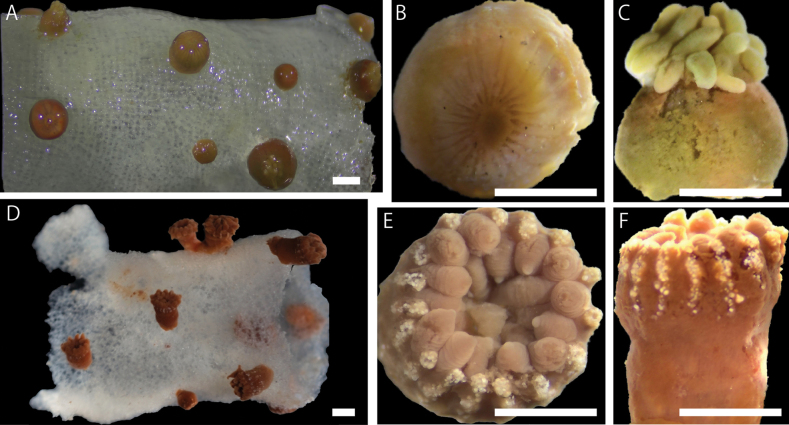
Images of external morphology of (**A–C**) *Vitrumanthusflosculus* sp. nov. and (**D–F**) *Churabanakuroshioae***A** preserved polyps attached to *Farrea* sp. **B** and **C** close-up image of a preserved polyp **D** preserved polyps attached to *Pararete* sp. **E** and **F** close-up image of a preserved polyp. Scale bars: 1.0 mm (**A–C**); 2.0 mm (**D–F**).

***External morphology*.** Colonial macrocnemic zoantharians associated with host hexasterophoran sponge *Farrea* Bowerbank, 1862 (Fig. 2A). Solitary or colonial polyps rise irregularly from all over the three-dimensional structure of host hexasterophoran sponge with base of polyps embedded in tissue of sponge (Fig. 3A). Preserved specimens consist of cylindrical polyps (Fig. 3B, C), dark brown in coloration. The living polyps and tentacles transparent yellowish in coloration. Surface of column smooth and ectoderm continuous (Fig. 3C). No encrustations of sand and silica particles in ectoderm of capitulum but ectoderm of scapus encrusted with small-sized sand and silica particles. Contracted preserved polyps 1.5–2.5 mm in height, 1.0–2.5 mm in diameter. Capitulary ridges indiscernible when contracted. Tentacles 22–26 in number.

**Figure 4.**
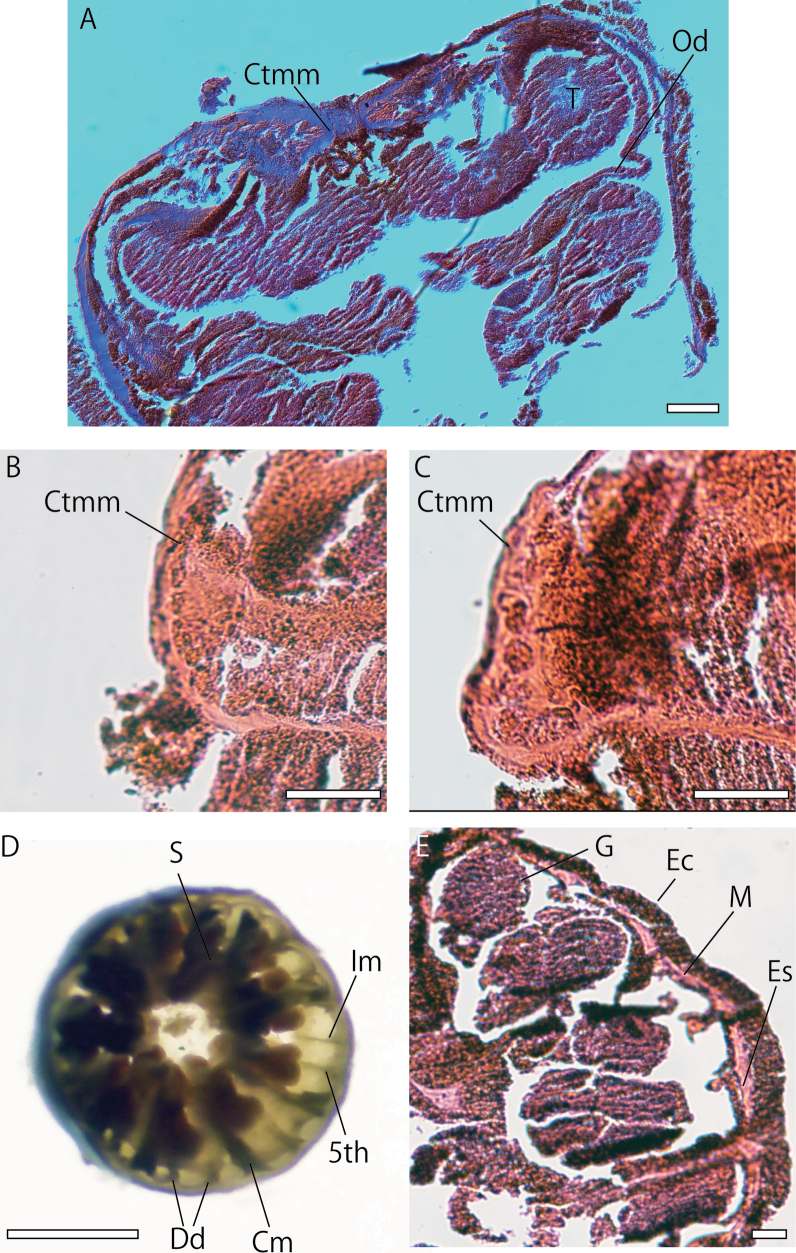
Images of internal morphology of *Vitrumanthusflosculus* sp. nov. **A** longitudinal section of a polyp **B** and **C** closed-up image of cyclically transitional marginal musculature **D** transverse-section of polyp at level of actinopharynx by hand-cutting **E** transverse-section of polyp. **Ctmm** cyclically transitional marginal musculature, **CM** complete mesentery, **Dd** dorsal directives, **Ec** ectoderm, **Es** encircling sinus, **IM** incomplete mesentery, **G** gonads, **M** mesoglea, **Od** oral disk, **T** tentacles, **S** siphonoglyph, **5^th^** 5^th^ mesentery from dorsal directives. Scale bars: 100 µm (**A**); 50 µm (**B, C, E**); 1 mm (**D**).

***Internal morphology*.** Zooxanthellae absent. Cyclically transitional marginal musculature (Fig. 4A–C). Encircling sinus or mesogleal canal present and basal canals of mesenteries absent (Fig. 4E). Mesenteries 22–26 in number, in brachycnemic arrangement (Fig. 4D). Mesoglea thickness 20–30 µm. Siphonoglyph distinct and V-shaped. Mesenterial filaments present. Complete mesenteries fertile (Fig. 4E).

**Figure 5.**
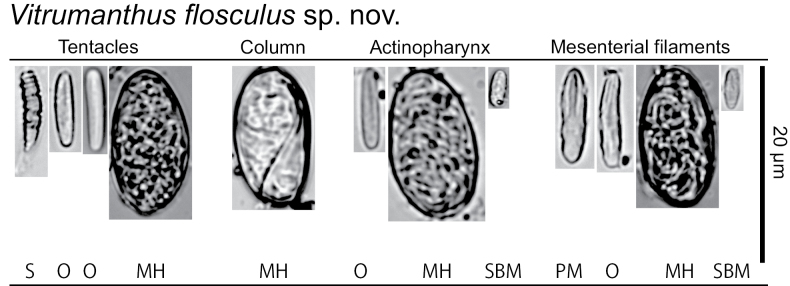
Cnidae in the tentacles, column, actinopharynx and mesenterial filaments of *Vitrumanthusflosculus* sp. nov. **HM** holotrich medium **O** basitrichs and microbasic b-mastigophores **PM** microbasic p-mastigophores **S** spriocysts **SBM** special microbasic b-mastigophores.

***Cnidae*.** Basitrichs and microbasic b-mastigophores, microbasic p-mastigophores, holotrichs, special b-mastigophores, and spirocysts (see Fig. 5, Table 1 for sizes and distributions).

**Table 1.** Cnidae types and sizes observed in this study. Frequency: relative abundance of cnidae type in decreasing order; numerous, common, occasional, rare. *N* = number of cnidae measured.

		*Vitrumanthusflosculus* sp. nov.			
Tissue	Type of cnidae	Length (min-max, mean)	Width (min-max, mean)	Frequency	n
Tentacle	Spirocysts	18.5–8.5, 12.7	3.3–1.3, 2.0	Numerous	215
	Basitrichs and microbasic b-mastigophores	12.4–8.5, 10.4	3.2–1.4, 2.1	Numerous	73
	Holotrichs (M)	20.5	9.7	Rare	1
Column	Holotrich (M)	20.9–17.0, 18.7	11.9–8.7, 10.0	Common	25
Actinopharynx	Basitrichs and microbasic b-mastigophores	18.2–8.7, 12.0	3.5–1.6, 2.3	Common	38
	Special microbasic b-mastigophores	8.6–7.0, 7.8	3.3–1.6, 2.3	Common	13
	Holotrichs (L)	19.7–13.0, 17.5	11.3–8.5, 10.2	Common	18
Mesenterial filaments	Bastrichs and microbasic b-mastigophores	14.7–7.9, 11.8	3.1–1.4, 1.9	Common	21
	Microbasic p-mastigophores	16.9–10.7, 13.2	3.7–2.5, 3.3	Common	32
	Special microbasic b-mastigophores	9.3–7.2, 8.1	3.0–2.1, 2.5	Occasional	5
	Holotrichs (M)	22.2–15.4, 18.6	11.3–7.6, 9.4	Common	25

##### Habitat and distribution.

Northwestern Pacific Ocean: known from the Shoho Seamount, Nishi-Shichito Ridge, Japan at a depth of 400 m. The new species was found on a glass sponge, *Farrea* sp., attached to rocks on the summit of the Shoho Seamount.

##### Associated host.

*Farrea* sp.

##### Remarks.

Regarding host sponges, *Vitrumanthusflosculus* sp. nov. is associated with *Farrea*, while other *Vitrumanthus* species are associated with other, different host sponges (*Vitrumanthusschrieri*: *Verrucocoeloidea*, *Parahigginsia* and *Cyrtaulon*, *Vitrumanthusvanderlandi*: *Aphrocallistes*, and *Vitrumanthusoligomyarius*: *Tretochone*). *Vitrumanthusflosculus* sp. nov. has holotrich nematocysts in all tissues we examined, while *V.vanderlandi* does not have holotrichs in any tissues. The surface of the column is smooth in *Vitrumanthusflosculus* sp. nov. with no encrustation of sand and silica particles in the ectoderm of capitulum, while the surface of the column is rough in *V.schrieri* with encrustation in the ectoderm of capitulum. The mesenteric arrangement of both *Vitrumanthusflosculus* sp. nov. and *V.oligomyarius* is brachycnemic, an exceptional characteristic for species within the suborder Macrocnemina. However, these two species can be distinguished by their numbers of tentacles and the sizes of the polyps; *Vitrumanthusflosculus* sp. nov. has 22–26 tentacles, while *V.oligomyarius* has 32–36 tentacles. *Vitrumanthusflosculus* sp. nov. has relatively smaller polyps than those of *V.oligomyarius* (1.5–2.5 mm in height and 1.0–2.5 mm in diameter vs. 0.5–3.1 mm in height and 1.2–3.4 mm in diameter). Furthermore, the host sponges of *Vitrumanthusflosculus* sp. nov. and *V.oligomyarius* are different (*Farrea* vs. *Tretochone*).

*Parachurabana* is a monotypic genus, and the diagnostic feature of this genus is described as having an association with Farreidae sponges. Although *Vitrumanthusflosculus* sp. nov. is associated with *Farrea* sp., *Vitrumanthusflosculus* sp. nov. can be easily distinguished from *Parachurbana* by the different shape of its sphincter muscle (cyclically transitional vs. cteniform endodermal marginal musculature) and different mesenterial arrangement (brachycnemic vs macrocnemic arrangement). The diagnosis of *Parachurabana* may need to be updated based on examinations of additional specimens.

#### ﻿Genus Churabana

Taxon classificationAnimalia

Kise, Montenegro & Reimer, 2021

A851B0FE-9CD0-5A73-8438-05E7F06730FF

##### Type species.

*Churabanakuroshioae* Kise, Montenegro & Reimer, 2021.

##### Diagnosis.

(modified from the diagnosis given by Kise et al. 2022). Parazoanthidae with obligate symbiotic relationship with *Pararete* sponges. Preserved polyps 3.0–10.0 mm in height, 2.8–5.0 mm in diameter. Azooxanthellate. Cteniform endodermal marginal musculature.

##### Remarks.

We modified the generic diagnosis based on a newly collected specimen of *Churabanakuroshioae.* This species seems to have host specificity to *Pararete* species based on this study and Kise et al. (2022), although further investigations are required to confirm this.

#### ﻿ Churabana kuroshioae

Taxon classificationAnimalia

Kise, Montenegro & Reimer, 2021

A29E3662-4B6C-5808-8A85-CE5C747CAB78

##### Material examined.

• NSMT-Co 1899, An’ei Seamount, Nishi-Shichito Ridge, Japan (29°17.03'N, 138°37.85'E), 770 m depth, October 17, 2021.

##### Type locality.

Near Iejima Island, Motobu, Okinawa, Japan.

##### Description.

External morphology. Parazoanthidae associated with host hexasterophoran sponge *Pararete Ijima*, 1927. Approximately 100 truncated cone-shaped cylindrical polyps in preserved specimen. Solitary or colonial polyps rise irregularly from host *Pararete* sponges (Figs 2B, 3D). The living and preserved polyps dark brown and tentacles brown in coloration. Ectoderm and mesoglea of capitulum encrusted with numerous and comparatively large sizes of sand and silica particles (approximately < 100 µm). No encrustations of sand and silica particles in the ectoderm or mesoglea of scapus (Fig. 3F). Contracted preserved polyps 3.0–10.0 mm in height, 2.8–5.0 mm in diameter. Capitulary ridges discernible when contracted, 15–16 in number, and 30–32 tentacles (Fig. 3E).

##### Habitat and distribution.

Northwestern Pacific Ocean: *Churabanakuroshioae* was originally reported from the Ryukyu Archipelago, Japan at depths of 520–650 m (Kise et al. 2022). The findings in this study reveal that this species is also distributed at the An’ei Seamount, Nishi-Shichito Ridge, Japan at a depth of 770 m. *Churabanakuroshioae* was found on the summit of An’ei Seamount on glass sponge *Pararete* sp. attached to rocky substrate.

##### Associated host.

*Pararete* sp.

##### Remarks.

The polyp coloration of *Churabanakuroshioae* is cream-pink or beige with cream or whitish transparent tentacles in the original description, while the specimen of *C.kuroshioae* collected from An’ei Seamount has dark brown polyps with brown tentacles. As well, the polyp sizes of the specimen examined in this study were relatively larger than that of the original description (3.0–4.0 mm in height, 2.8–4.0 mm in diameter) by Kise et al. (2022). Based on the results of molecular phylogenetic analyses, the differences in coloration and polyp sizes found in this study are considered intraspecific variation, although detailed molecular analyses in the future may warrant reconsideration of this.

### ﻿Mitochondrial genome

The complete mitochondrial genome sizes of *Churabanakuroshioae* and *Vitrumanthusflosculus* sp. nov. were 22,738 and 20,556 bp, respectively. The mitochondrial gene order and content of these two species were the same, including 13 protein-coding genes, two rRNA genes, and two transfer RNA genes. The sequences of the protein-coding region covered 54.0% (*Churabanakuroshioae*) and 58.8% (*Vitrumanthusflosculus* sp. nov.) of the mitochondrial genomes, while GC contents of *Churabanakuroshioae* and *Vitrumanthusflosculus* sp. nov. were 49.8% and 50.0%, respectively. Regarding stop codons, both *C.kuroshioae* and *V.flosculus* sp. nov. have either TAA and TAG for all protein-coding genes, with the start codon being ATG. The mitochondrial base composition was A: 22.6%, T: 27.6%, G: 26.1%, C: 23.7% in *C.kuroshioae*, and A: 22.2%, T: 27.8%, G: 26.3%, C: 23.6% in *V.flosculus* sp. nov.

### ﻿Molecular phylogeny

**Figure 6.**
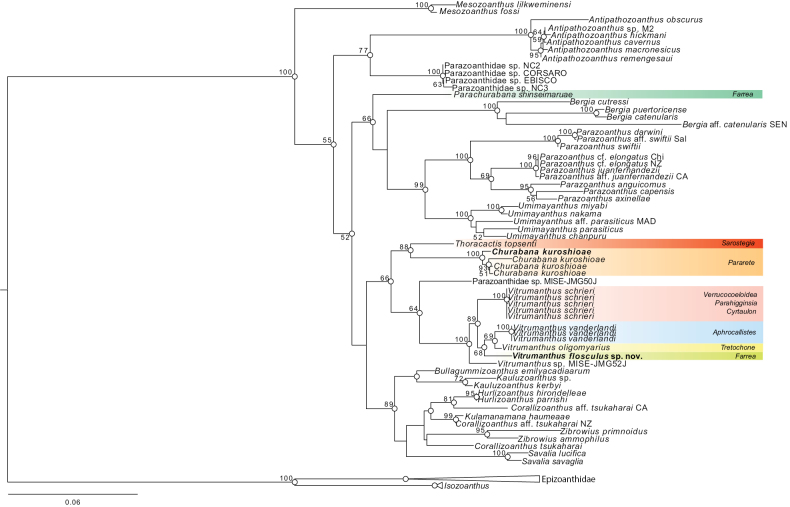
Maximum-likelihood tree based on combined dataset of CoxI, 12S rDNA, 16S rDNA, 18S rDNA, 28S rDNA, and ITS rDNA sequences. Numbers at nodes represent ML bootstrap values (>50% are shown). White circles on nodes indicate high support of Bayesian posterior probabilities (PP) (>0.95).

ML and BI phylogenetic analyses based on the six-gene dataset indicated that *Churabana* and *Vitrumanthus* were both monophyletic clades with complete support (ML = 100%, BI = 1). *Churabana* was sister to *Thoracactis* (ML = 88%, BI = 0.96). ML and BI phylogenetic topologies were congruent (Fig. 6). *Vitrumanthusflosculus* sp. nov. was sister to *Vitrumanthusoligomyarius* and *Vitrumanthusvanderlandi* (ML = 68%, BI = 0.99).

**Figure 7.**
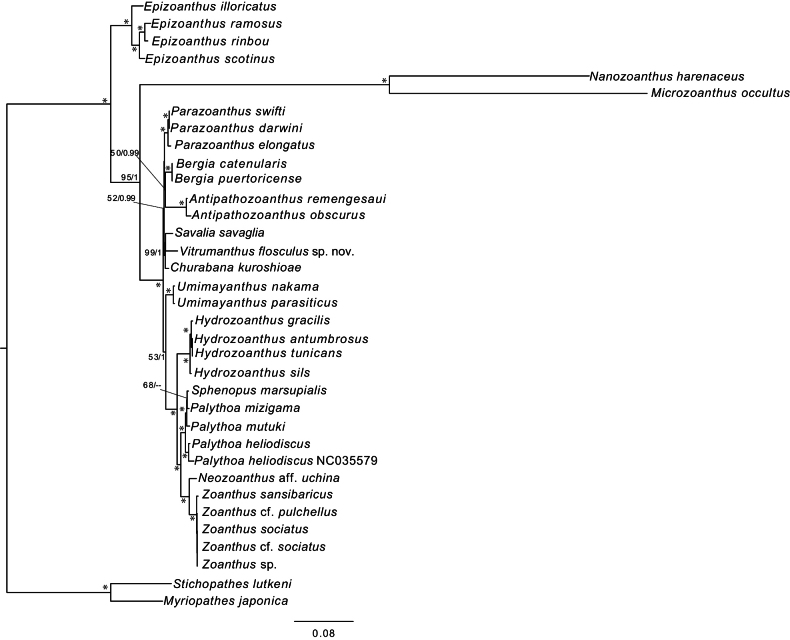
Maximum-likelihood tree based on complete mitochondrial genome dataset. Numbers at each node indicates ML bootstrap value and Bayesian posterior probabilities. Asterisks indicate ML/BI = 100/1.00.

ML and BI phylogenetic topologies based on the complete mitochondrial genome dataset were also congruent (Fig. 7). *Churabana* and *Vitrumanthus* formed a monophyletic clade with *Savaliasavaglia* (Bertoloni, 1819) with strong support (ML = 99%, BI = 1).

## ﻿Discussion

*Thoracactistopsenti* was the first zoantharian species to be described as Hexasterophora-associated (Gravier 1918). Subsequent studies have more recently described three Parazoanthidae genera associated with hexasterophorans from the Indo-Pacific and the Atlantic (Kise et al. 2022, 2023). Although *Thoracactis* was originally placed in the family Epizoanthidae, Kise et al. (2024) have recently transferred *T.topsenti* to Parazoanthidae based on molecular phylogenetic and morphological results, indicating that the association with Hexasterophora is unique to the family Parazoanthidae. Kise et al. (2023) found that Hexasterophora-associated species were not monophyletic, but instead that *Parachurabana* was recovered as basal to Demospongiae-associated species (*Bergia*, *Parazoanthus*, and *Umimayanthus*), indicating that Parazoanthidae species may have switched its host from Hexasterophora to Demospongiae. However, the phylogenetic tree based on a six-gene dataset of this study and previous studies have shown weak support at some nodes in Parazoanthidae. Therefore, further studies using phylogenetically informative loci, as mentioned below, are needed to better understand the evolutionary history of host switching in Parazoanthidae.

This study sequenced the complete mitochondrial genomes of *Churabana* and *Vitrumanthus* for the first time. The mitochondrial gene arrangements of these two genera were in the same order as those of other zoantharians (Poliseno et al. 2020), further reinforcing the conservative nature of zoantharian mitochondrial gene orders.

Based on both our six-gene and complete mitochondrial genome analyses, it is apparent that much of the diversity of Parazoanthidae has comparatively recently evolved, resulting in weak support at many generic–level nodes, with short genetic distances as reported in Poliseno et al. (2020). Perhaps more robust genomic analytical methods (e.g., ultra-conserved elements; Cowman et al. 2020; Quattrini et al. 2020) may help resolve the weak phylogenetic structure of Parazoanthidae, which would then help in taxonomic reconsideration of the family. Most of the genera contained within Parazoanthidae have been erected since 2008 (12/17 genera), with each genus erected based on its uniqueness from other genera, and little consideration has yet been given to the phylogeny and taxonomy of the family. It may be time to reconsider the framework of Parazoanthidae, and it is hoped that the current study provides the impetus to begin this future work.

Shoho and An’ei seamounts are on the Nishi-Shichito Ridge, which has been designated as a marine protected area (MPA) (Ministry of the Environment of Japan 2020), and recent studies have described a number of previously unknown species including sea pens, sea stars, ribbon worms, and parasitic crustacean from the Shoho and An’ei seamounts (Hookabe et al. 2021, 2023; Kobayashi et al. 2022; Jimi et al. 2023; Kushida et al. 2024). Our results echo these recent studies, highlighting the overall lack of diversity studies in this MPA. Documentation of local faunal biodiversity is one important key for effective monitoring of MPAs, and further taxonomic studies of many taxa are needed to better understand the true marine diversity of this MPA.

## Supplementary Material

XML Treatment for Vitrumanthus

XML Treatment for Vitrumanthus flosculus

XML Treatment for Churabana

XML Treatment for Churabana kuroshioae

## ﻿Additional information

### Author ORCIDs

Hiroki Kise https://orcid.org/0000-0002-4099-6469

James Davis Reimer https://orcid.org/0000-0003-0453-8804

Akira Iguchi https://orcid.org/0000-0002-8894-1977

Yuji Ise https://orcid.org/0009-0009-4887-611X

Shinji Tsuchida https://orcid.org/0000-0002-8600-2386

Yoshihiro Fujiwara https://orcid.org/0000-0002-1833-1866

### Data availability

All of the data that support the findings of this study are available in the main text or Supplementary Information. Obtained sequences have been deposited in NCBI GenBank (accession number PQ308072–PQ308077 and PQ554681–PQ554682).

## Supplementary materials

Supplementary material 1 GenBank accession numbers used for phylogenetic analyses in this study Data type xlsx https://binary.pensoft.net/file/1208604This dataset is made available under the Open Database License (http://opendatacommons.org/licenses/odbl/1.0/). The Open Database License (ODbL) is a license agreement intended to allow users to freely share, modify, and use this Dataset while maintaining this same freedom for others, provided that the original source and author(s) are credited.Hiroki Kise, James Davis Reimer, Akira Iguchi, Yuji Ise, Shinji Tsuchida, Yoshihiro Fujiwara

Supplementary material 2 Best fitting models for ML and BI phylogenetic analyses based on six-gene dataset Data type xlsx https://binary.pensoft.net/file/1208605This dataset is made available under the Open Database License (http://opendatacommons.org/licenses/odbl/1.0/). The Open Database License (ODbL) is a license agreement intended to allow users to freely share, modify, and use this Dataset while maintaining this same freedom for others, provided that the original source and author(s) are credited.Hiroki Kise, James Davis Reimer, Akira Iguchi, Yuji Ise, Shinji Tsuchida, Yoshihiro Fujiwara

Supplementary material 3 Information of Zoantharian species used for phylogenomic analyses of mitochondrial genomes Data type xlsx https://binary.pensoft.net/file/1208606This dataset is made available under the Open Database License (http://opendatacommons.org/licenses/odbl/1.0/). The Open Database License (ODbL) is a license agreement intended to allow users to freely share, modify, and use this Dataset while maintaining this same freedom for others, provided that the original source and author(s) are credited.Hiroki Kise, James Davis Reimer, Akira Iguchi, Yuji Ise, Shinji Tsuchida, Yoshihiro Fujiwara

Supplementary material 4 Best fitting models for ML and BI phylogenetic analyses based on complete mitochondrial genome dataset Data type xlsx https://binary.pensoft.net/file/1208607This dataset is made available under the Open Database License (http://opendatacommons.org/licenses/odbl/1.0/). The Open Database License (ODbL) is a license agreement intended to allow users to freely share, modify, and use this Dataset while maintaining this same freedom for others, provided that the original source and author(s) are credited.Hiroki Kise, James Davis Reimer, Akira Iguchi, Yuji Ise, Shinji Tsuchida, Yoshihiro Fujiwara
